# EFFECT OF COCHLEAR IMPLANT ON GAIT AMONG ADULTS WITH AGE-RELATED HEARING LOSS: A SYSTEMATIC REVIEW

**DOI:** 10.1093/geroni/igae098.1209

**Published:** 2024-12-31

**Authors:** Bahaa Rafoul, Maayan Agmon, Hanin Karawani

**Affiliations:** University of Haifa, Haifa, Hefa, Israel; University of Haifa, University of Haifa, HaZafon, Israel; University of Haifa, Haifa, Hefa, Israel

## Abstract

Research shows a link between hearing loss, gait impairment, and an increased fall risk. A number of reviews on the effect of hearing interventions on balance have been published; however, whether Cochlear Implant (CI) can improve gait impairment and reduce fall risk remains unclear. This systematic review evaluates the impact of CIs on the walking abilities of adults with hearing loss. It contributes to the theoretical understanding of the link between hearing loss and gait, as well as to the clinical understanding of the combined effect of treatment on gain improvement. Utilizing PRISMA guidelines, we extracted seven relevant studies from PubMed, Web of Science, and Scopus. Five studies included CI users, while two included CI and hearing aid users. Inconsistency in measurement methodologies and follow-up durations were noted, contributing to incongruent findings. We found that a short follow-up (up to two months) after the activation of the CI was associated with non-improved and even worsening gait, and a longer follow-up (at least three months) was associated with a partially improved gait. However, due to various methodological inconsistencies in the seven articles available, it remains challenging to fully understand the effects of hearing treatment by the CI on gait. We suggest that future research should consider underlying mechanisms such as cognition and function in dual-task walking with longer follow-up to better understand the link between hearing and gait, especially after hearing intervention.

